# Distribution and Determinants of Serum Zinc, Copper, and Selenium Levels among Children under Five Years from Popokabaka, Democratic Republic of Congo: A Cross-Sectional Study

**DOI:** 10.3390/nu14030683

**Published:** 2022-02-06

**Authors:** Branly Kilola Mbunga, Ingunn M. S. Engebretsen, Tor A. Strand, Elin L. F. Gjengdal, Pierre Z. Akilimali, Mina M. Langfjord, Pierrot L. Tugirimana, Mala Ali Mapatano

**Affiliations:** 1 Kinshasa School of Public Health, University of Kinshasa, Kinshasa, Congo; pierretulanefp@gmail.com (P.Z.A.); mapatanow@yahoo.fr (M.A.M.); 2 Department of Global Public Health and Primary Care, Centre for International Health, University of Bergen, 5009 Bergen, Norway; Ingunn.Engebretsen@uib.no (I.M.S.E.); tor.strand@uib.no (T.A.S.); 3 Faculty of Environmental Sciences and Natural Resource Management, Norwegian University of Life Sciences, 1432 Ås, Norway; elin.gjengedal@nmbu.no (E.L.F.G.); mina.marthinsen.langfjord@nmbu.no (M.M.L.); 4 Faculty of Medicine, University of Goma, Goma, Congo; pltugirimana@gmail.com

**Keywords:** zinc, copper, selenium, children, Popokabaka, deficiency

## Abstract

Information about essential trace elements among children in many African countries, including the Democratic Republic of Congo (DRC), is limited. We aimed to measure the distribution and determinants of serum zinc (Zn), copper (Cu), and selenium (Se) concentrations in a representative sample of children under five years old. We conducted a community-based cross-sectional study in Popokabaka, DRC. Blood samples were drawn from 412 children. The serum concentrations of minerals were measured using inductively coupled plasma–mass spectrometry. The median concentrations (P25–P75) of Zn, Cu, and Se were 61.9 µg/dL (52.8–70.2), 145.5 (120.0–167.0) µg/dL and 5.3 (4.3–6.3) µg/dL. The CRP-adjusted prevalence of serum Se deficiency was 84.1% (95% confidence interval [CI] 81.4–87.0) and of Zn deficiency was 64.6% (95% CI 59.8–69.1%). Only a few children were Cu deficient [1.5% (0.6–3.2)]. Evidence of inflammation (C-reactive protein, >5 mg/L) was associated with a lower Se concentration and higher Cu concentration. Furthermore, serum Se concentration was positively associated with linear growth. The average Cu/Zn molar ratio (2:1) was twice that recommended. Children in western Popokabaka had higher Zn and Se levels than their eastern neighbors. Zinc and selenium deficiencies are common among children in Popokabaka and require attention and prioritization.

## 1. Introduction

Zinc (Zn), copper (Cu), and selenium (Se) are essential minerals [1] because they generally serve as modulators in various chemical, biological, catalytic, and metabolic processes [2], and play a crucial role in optimal growth and cognitive development during childhood [3]. Furthermore, they are all critical co-factors for the immune system and function in antioxidant-stress modulation. In addition, Zn specifically stabilizes the DNA structure and mediates its replication [4]. Selenium plays a specific role in thyroid function, and its deficiency is associated with thyroid diseases [5]. Copper is involved in erythrocyte differentiation, and its deficiency may cause hypochromic microcytic anemia [6].

As nutrients, they are obtained from food, water, and the environment [7] and are usually needed in tiny quantities (traces) in the body [8,9]. Their toxicities are rare at the dietary intake level, as they are highly regulated by homeostatic mechanisms to avoid excessive levels and cannot be stored in the body [1]. Deficiencies result from inadequate dietary intake, malabsorption, or other conditions causing intestinal loss [10]. According to the World Health Organization (WHO), deficiencies of these minerals are widespread and contribute to childhood morbidity and mortality, mainly through impaired immunity [11,12].

Although regional projections [13,14,15] predict the severity of deficiencies of the essential trace elements (TEs) in the sub-Saharan regions, documented information on serum Zn, Cu, and Se concentrations in children from this region is limited. Moreover, the national-level data are limited, probably due to their perceived high cost and logistical challenges [15]. In addition, the recent recognition of inflammation’s role in influencing mineral balance [16,17,18,19] limits their accurate interpretation, particularly in populations with a high burden of infections. Thus, nutritional assessment of essential minerals should consider exploring inflammatory health conditions affecting the concentrations [20].

In the Democratic Republic of Congo (DRC), reliable information on the burden of Zn, Cu, and Se deficiencies in the normal population is limited. Harvey-Leeson et al. reported a high prevalence of Zn deficiency among children of the Kivu and Kongo Central provinces [21]. Musimwa et al. [22], in the Lubumbashi region, compared the TE levels of severely malnourished children and well-nourished ones, and found that the former had lower TE levels. Bumoko et al. [23], in the rural area of Kahemba, found much lower serum Se, Zn, and Cu levels in Konzo children, and that Se status was positively associated with neurodevelopment.

Considering the unchanged stunting trend and the high infant morbidity and mortality rates in the Democratic Republic of Congo, factors that can impair health and growth, such as Zn, Cu, and Se status in a representative sample of children, should be identified. Therefore, this study aimed to describe the serum Zn, Cu, and Se concentrations and identify their determinants among the children in Popokabaka, DRC.

## 2. Materials and Methods

### 2.1. Design and Study Location

The present analysis is part of a population-based cross-sectional study conducted between May and June 2019 in the Popokabaka health zone, Kwango Province (formerly Bandundu Province), DRC. This region (5°22′49.26″ S–5°5′48″ S, 16°20′26.16″ E–16°22′14.88″ E) is known for the poor soil and food insecurity. It is an entirely rural region without a known mining history, but is close to the Kahemba region and Angola border, known for their notorious diamond mining exploitation. The Kwango River, which takes its source in Angola and crosses the mining region, divides the Popokabaka region into two. Agriculture, based on monoculture, remains the primary source of income for the community. People prefer to grow cassava as their staple food because of its resistance to soil drought, marketable roots, and culturally accepted leaves. Konzo, a neurotoxic motor disease, is prevalent in the region, mainly affecting children and women [15,16,17,18]; it is a disease explained by incomplete preparation of bitter cassava. The people in Popokabaka have limited access to agricultural fertilizers. Livestock and poultry are less practiced due to the lack of drugs to fight epidemics in animals. People avoid fishing due to their fear of crocodiles in the Kwango River. Animal-sourced foods are, thus, rarely consumed, and accessibility for imported foods and goods from other towns is also limited because of poor roads. People in Popokabaka drink untreated water from groundwater sources. Chronic malnutrition is common because half of all children are stunted [24,25]. Malaria and anemia are also highly prevalent [26].

### 2.2. Participants and Sampling

A total of 432 children aged 12–59 months were included in this study. As the present research is part of a multiple-outcomes biomarker survey, the calculation was based on a proportional sample size calculation for anemia prevalence of0.59, a precision of 0.075, and a design effect of two. Children were selected using a three-stage cluster sampling technique. More details on sampling are described in our former article [26]. 

**Figure 1:**
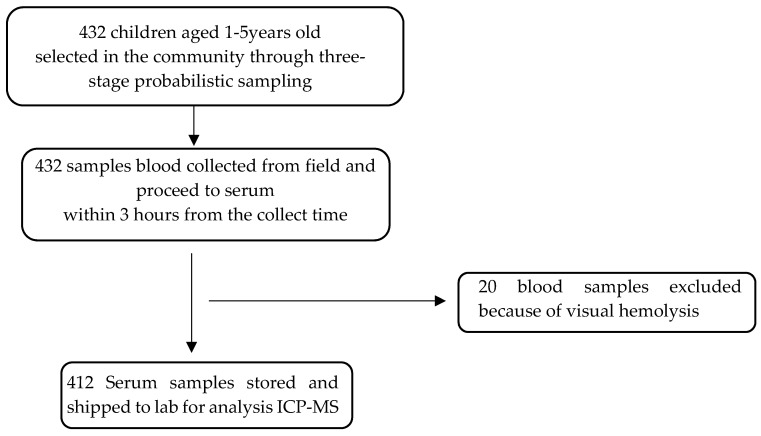
Flowchart of participants recruitment.

The flow chart in Figure 1 shows the recruitment and sample exclusion conducted in the present study. Children belonged to five health areas: Kabangu, Ingasi, Cite Popo, Secteur Popo, and Tzunza. Four of them are on the eastern side, and one is on the western side of the Kwango River.

### 2.3. Data Collection Technique

Data collection was organized in 2 consecutive days in each cluster: a household survey with anthropometry on the first day and blood collection on the following day. More details can be found in our previous paper [26]. Anthropometric indices including weight-for-height, height-for-age, weight-for-age, and mid-upper-arm-circumference-for-age, and their Z Scores, were calculated using WHO Anthro software. 

### 2.4. Blood Processing and Management

On the second day, phlebotomists first performed a capillary finger-prick test for hemoglobin (Hgb) assessment (Hemocue 301) and a rapid test for malaria in the field. Then, they collected up to 6 mL of venous blood from 432 children aged 1–5 years. The serum was separated from the blood cells within three hours. We used trace-element-free equipment and techniques previously described to ensure that samples were not contaminated [26]. We excluded blood samples (*n* = 20) with visual hemolysis or insufficient quantity. The separation was performed at 2300 rpm for 10 min (RCF 1532 g) using a Hettich centrifuge (Tuttlingen, Germany). The serum was aliquoted into two polypropylene vials: 0.5 mL tricoded FluidX vials (Brooks Life Science) and 2 mL Sarstedt vials (Sarstedt, Nümbrecht, Germany). We stored the vials, frozen at −40 °C, until the survey ended. Then, we transported all samples in liquid nitrogen from Popokabaka to Kinshasa (a 12 h vehicle trip). The vials were transferred to −80 °C ultra-low freezers at Kinshasa School of Public Health for a week. Then, vials were shipped on dry ice and under continuous temperature monitoring to two labs in Norway. All of the 0.5 mL vials were sent to Haukeland University Hospital (Bergen, Norway) for the analysis of serum ferritin (using an electrochemiluminescence immunoassay (ECLIA)), C-reactive protein (S-CRP) (using the immunoturbimetry method), and total iron-binding capacity (S-TIBC, using the Berekna equation calculation: s-TIBC = s-Transferrin * 25.1). Two-milliliter vials were sent to the Norwegian University of Life Sciences (Ås, Norway) for the analysis of Zn, Cu and Se (using the Agilent 8900 Triple Quadrupole inductively coupled plasma–mass spectrometer (ICP-MS)). Transferrin saturation (TSAT), expressed as a percentage, was then calculated as the value of serum iron divided by the TIBC.

### 2.5. Sample Preparation for Mineral Analysis

Using a 100–1000 µL pipette (Sartorius, Göttingen, Germany) and Thermo Scientific ART Barrier pipet tips (Waltham, MA, USA), 250-µL aliquots of thawed, tempered, and homogenized serum was transferred into 5 mL polypropylene tubes (Sarstedt, Nümbrecht, Germany) and accurately weighed (Sartorius MC 210P). Subsequently, using a 10–300 µL electronic pipette (Biohit, Helsinki, Finland), 100 µL of internal standard (rhodium [Rh] and selenium [74Se]) and 500 µL of nitric acid (HNO3, 69% weight [w]/w, sub-boiled ultra-pure) were added to each sample before digesting for 3 h at 90 °C in a heating cabinet (Termaks, Bergen, Norway). Finally, the samples were diluted to 5.00 mL with deionized water (>18 MΩ). To stabilize mercury in the solution, 100 µL hydrochloric acid (HCl, 37% *w*/*w*, sub-boiled ultra-pure) was added to each sample.

### 2.6. Sample Analysis

The total element concentrations in the serum were quantified by inductively coupled plasma–mass spectrometry using the Agilent 8900 Triple Quadrupole (QQQ) ICP-MS. The masses were (Q1/Q2): Cu (63/63) and Zn (66/66) with gas-mode ammonia (NH3) and Se (78/94), using gas-mode oxygen (O2). Detection and quantification limits were calculated by multiplying the standard deviations of the blank samples (*n* = 10) by three and ten, respectively. The ten blank samples were taken through the whole measurement procedure, including the sample preparation steps. The limit of detection (LOD) and limit of quantification (LOQ) were determined in µg/dL as Cu (0.1/0.49), Zn (6/20), and Se (0.08/0.27), respectively.

### 2.7. Quality Control

We assessed the blank samples for contamination of reagents and the equipment used. The accuracy was evaluated by concurrent analysis of SeronormTM Trace Elements, Serum L1 and L2 (Billingstad, Norway). The data obtained were within a 95% confidence level of the certified values issued. The method’s “within-laboratory reproducibility” (RSD) was <1.4% for Zn, Cu, and Se; the results were obtained by measuring 12 replicate samples of the serum on three different days.

### 2.8. TE Threshold Definitions

We defined deficiency at a level of Cu <80 µg/dL, Zn <65 µg/dL [27], and Se <7.0 µg/dL [10]. Anemia was defined as Hgb levels <11 g/dL, and Iron Deficiency was set for transferrin saturation <20%. To account for inflammation [28], the regression-correction approach developed by BRINDA was used for minerals that correlated with CRP using the following equation: Adjusted mineral = unadjusted mineral—β (CRPobs–CRPref). First, we defined internal reference values for inflammatory markers (CRPref) as the tenth percentile [29]. Then, the regression coefficient (β) for the association was estimated between CRP and each mineral value using univariable linear regression models, with the minerals as dependent variables.

### 2.9. Statistical Management

Data were analyzed using Stata 16.1 (StataCorp LLC, College Station, TX, USA). First, we investigated the normal distribution of TE concentrations using the Kolgomorov–Smirnov test. None of TEs were normally distributed. Statistics were then summarized as medians with 25th and 75th percentiles (P25–P75). The prevalence was reported as proportions with their 95% confidence intervals (95 CI). We also calculated the Cu/Zn ratio. Then, the Mann–Whitney and Kruskal–Wallis tests were performed to compare mineral concentrations across categorical variables, whereas Spearman’s rank–order test coefficient was used to assess any correlation among continuous variables. A simple linear regression analysis was used to adjust mineral concentrations for inflammation (CRP). Bivariate and multiple linear regression analyses were performed to identify significant determinants for every mineral. Then, we reported the unadjusted and adjusted regression coefficients, 95% CI, and corresponding *p*-values. The final regression models were constructed following a forward stepwise selection approach with covariate inclusion probability (*p*-value of crude odds ratio) of <0.20. Multicollinearity, moderation and mediation effects were checked for all of the regression models.

## 3. Results

### 3.1. Characteristics of the Study Population

The serum was collected from 412 children aged 1–5 years. The boy/girl ratio was 1:1, and their median (P25–P75) age was 32 [22,23,24,25,26,27,28,29,30,31,32,33,34,35,36,37,38,39,40,41,42,43] months. Table 1 shows the general characteristics of the children included in this study. Stunting was the most common nutritional problem observed in 55.3% of the children, whereas one in ten children (10.7%) was wasted. From the two preceding weeks, half of the children (58.0%) had experienced symptoms such as diarrhea (17.2%) and cough (32.3%). This inflammatory status was also confirmed in 49% of the children who had an elevated CRP level. Anemia was prevalent in 68% of the children, and iron deficiency was not equally common (7.5%). Under this tropical location, malaria was the leading health problem, and was found in nine in ten children (86.9%). Many of the children had received nutritional supplements, such as Zn (5.1%) and iron (35.7%), two weeks before the study initiation.

### 3.2. Distribution of Serum Zn, Cu, and Se

The serum Zn, Cu, and Se concentrations are shown in Table 2 as medians (P25–P75). The concentrations of these nutrients were not associated with age or sex. Acute inflammation status was negatively associated with Zn and Se levels, and positively associated with Cu levels. Children with elevated CRP levels (>5) had lower Zn and Se levels than those with normal CRP levels (*p*-value < 0.001). In contrast, children with elevated CRP levels had higher Cu levels than those with normal Cu levels (*p*-value < 0.001). Among the three minerals, only Se was associated with stunting and anemia. Children with stunting (HAZ Z Score < –2) or anemia (Hgb < 11 g/dL) were more likely to have a more profound Se deficiency. Having malaria was not associated with any of the micronutrient concentrations.

Considering that the Kwango River completely halves the Popokabaka health zone, children from villages on the eastern side of the river were found more deficient in Se and Zn than those from villages on the western side.

### 3.3. Prevalence of Deficiencies of Zn, Cu, and Se

Zn and Se deficiencies were widespread among children in Popokabaka (see Table 3), whereas Cu deficiency was found only in 6 of the 412 (1.5%) children. Se deficiency prevalence was severe in 86.9% (95 CI 83.3–89.8) of the children, without considering inflammation. Inflammation adjustment lowered the Se deficiency prevalence to 84.1 % (81.4–87). Zn deficiency was observed in 64.6% (95 CI 59.8–69.0) of the children. 

### 3.4. Correlations between the Biomarkers

Bivariate correlations between the different biomarkers are shown in Table 4; this table reveals that the serum Zn levels were positively correlated with serum Cu and Se levels. Simultaneously, no relationship was observed between the serum Cu and Se levels. The Cu/Zn ratio, known for its association with cognitive functioning in early life, scored 2:1, twice more the standard accepted. Second, the serum Zn and Se levels were positively correlated with hemoglobin levels, whereas Cu was negatively correlated with transferrin saturation (TSAT). In other words, the lower the Zn or Se level in children, the lower the hemoglobin level. Moreover, the higher the Cu level, the higher the occurrence of iron deficiency. Finally, Zn and Se concentrations were negatively correlated with CRP level, whereas Cu was positively correlated with CRP.

### 3.5. Determinants of Serum Zn, Cu, and Se

The Table 5 is showing determinants of serum Zn, Cu, and Se concentrations. Regarding Zn, only the side of Kwango River on which children were located determined the serum Zn levels. Children living in villages on the western side had higher serum Zn than those from the eastern side (increased by 5.95). Regarding Cu, the inflammatory state was a powerful determinant that positively determined Cu levels in the children of Popokabaka. With inflammation (CRP ≥ 5 mg/dL), the serum Cu level increased by 23 μg/dL. Transferrin saturation negatively determined the Cu level, indicating that the more iron deficient (TSAT decreasing) the child was, the higher his/her Cu level was. Conversely, children with higher Cu levels had been administered iron tablets in the two previous weeks. Girls had less Cu than boys. Selenium was positively related to the height-for-age Z Score, indicating that the more stunted the child was, the more Se-deficient he or she was. Consistent with Zn, serum Se was more concentrated in children on the left side of Popokabaka. Inflammation negatively determined the Se level, even after adjustments.

## 4. Discussion

The present study was conducted in the context that there is a lack of community-based data on serum levels of essential TEs. In a representative sample of children aged <5 years from the Popokabaka region, we assessed serum Zn, Cu, and Se levels and identified determinants of these minerals. In short, Zn and Se deficiencies were severely prevalent, and normal Cu levels were observed, implying a mineral imbalance (Cu/Zn ratio as 2:1) potentially contributing to cognitive development and growth impairments. The study also determined some factors that possibly explain the variation of serum Zn, Cu, and Se levels, and that could guide decisions and policy making.

### 4.1. Zn Deficiency

Zn is necessary for adequate growth, immunocompetence, and cognitive development [30,31]. Using Zn availability indicators in the food supply database and a stunting indicator, Wessells et al. [15] predicted a severity (>40%) of Zn deficiency risk for the DRC region. Accordingly, 64% of children were Zn deficient in our study. Children of Popokabaka lack Zn, like children in other sub-Saharan countries [14]. However, our observations in Popokabaka (Kwango Province) seem to be more severe than the ones from the Kongo Central and south Kivu provinces in DRC (25–29%), as reported by Harvey-Leeson in 2016 [21]. This remarkable difference could be explained by the difference in dietary habits and the better food availability and diversity observed in the Kongo Central and south Kivu regions than in Popokabaka [32]. For instance, communities in the Kivu province are pastoral, commonly practice livestock, and have great accessibility to meat, beans, and potatoes, which are culturally accepted as staple foods. The Kongo Central province also has varied food availability because of its geopolitical situation as the entrance for food importation from the Atlantic Sea. Thus, dietary customs are diversified with possible high Zn intake in these communities. By contrast, Popokabaka is enclaved, entirely rural, hard to reach, and almost inaccessible for food importation from big towns in DRC. Crop variety and animal-sourced foods are limited, and poverty hurts the population, as evidenced by the high level of Se and Zn deficiency. As is already known, food accessibility contributes to increasing dietary diversity and nutritional intake in a community [33]. These studies imply high variation and disparities in serum Zn deficiency across DRC provinces, and further national-scale surveys should be considered in DRC. Moreover, location remained the only stable determinant in our Zn model. Those living on the western side of the Kwango River had higher serum Zn levels. This reinforces the hypothesis of geographical disparities of Zn across regions, although the spatial analysis was not performed to confirm the spatial dependency of Zn deficiency, as established in the literature [34,35].

The negative effect of inflammation status on serum Zn levels observed in bivariate analysis comes to null after adjusting other covariates in the multivariate regression model. This observation is in discordance with the literature that supports a stable negative determination of inflammation [16,21,36,37,38]. We know that in inflammatory conditions, Zn moves from plasma to the liver in the production of pro-inflammatory molecules, lowering its plasma or serum level. This effect was suppressed with our data, probably because only the acute phase was considered using the CRP as a biomarker. We did not measure the Alpha-globulin glycoprotein, which better reflects chronic inflammation, as there would be a classification bias that distorted the true expected effect. Again, Zn was not determined by inflammatory-related diseases (diarrhea or cough in the last two weeks) in our data, although other studies indicate a reduction effect of zinc on morbidity and mortality for childhood diarrhea and pneumonia [39,40,41,42] and adult COVID-19 [43]. We hypothesize that other Zn influencing factors (Zn dietary intake, Zn absorption, and the ratio of phytate/Zn) should be considered in a further Zn model. In addition, unpublished documentation from Popokabaka health management reports high diarrhea incidence, justifying the systematic use of Zn supplements as an adjunct therapy for diarrhea in Popokabaka health facilities.

As previously observed, stunting prevalence, an indirect indicator of Zn deficiency [10], is also severe in Popokabaka. The coexistence of severe stunting, Zn deficiency, and Se deficiency in Popokabaka suggests stable, permanent, and long-term essential TE deficiencies that require specific food-based strategies [44].

Zn is known to contribute to growth. However, we found no association with the height-for-age Z Score. This observation is also in line with several observational studies [45,46,47,48,49,50]. The serum Zn level is contextual, changing, and highly influenced by the moment and dietary intake, and may not be explained by stature retardation, which has occurred in the early stages of life. Moreover, the cross-sectional design does not allow any prospective assessment of Zn and growth.

### 4.2. Cu Deficiency, Cu-Deficiency Anemia, and Cu/Zn Ratio

The present study reported a normal serum Cu concentration among children of Popokabaka, with 1.5% Cu deficiency prevalence, without being related to anemia [39], but a negative correlation with transferrin saturation. This imbalance may result from increased circulating ceruloplasmin as a stress response, resulting from inadequate hemoglobin synthesis or inflammation. Ceruloplasmin, also known as ferroxidase, contributes to the homeostatic regulation of both minerals in the serum. It binds to approximately 95% of serum Cu and oxidizes ferrous iron [3].

Cu, a bivalent mineral, is also an antagonist Zn. A Zn shift from the plasma into the liver during the inflammation response tends to increase Cu as plasma Zn decreases. Other studies [51] also reported such a mineral imbalance and normal Cu distribution with high Zn deficiency. In addition, the Cu/Zn ratio is a valuable parameter for a range of detrimental health conditions: increased Cu/Zn ratio may indicate growth impairment, cognitive abnormalities, risks of bowel diseases, and increased oxidative stress and cardiovascular disease [18,52]. We reported a Cu/Zn ratio of 2:1, which was twice the recommended standard (1:1), implying a need for an urgent Zn strategy (Zn fortification or supplementation) to increase Zn, thereby correcting the Cu/Zn ratio among children of Popokabaka.

### 4.3. Se Deficiency

Considering a lesser-known nutrient, Se plays a role in DNA reproduction, metabolism, synthesis, and protection from oxidative damage caused by free radicals [3]. For the first time in the DRC, the present study reported the prevalence of serum Se deficiency from a representative population of children. Eight in ten children in Popokabaka (exactly 84.1%) had Se deficiency. This severity is consistent with the prediction of Se risk deficiency (76–90%, for DRC) based on indirect indicators (Se supplies in food systems) for the sub-Saharan regions, reported by Joy et al. in 2014 [53]. Humans take Se from the soil and water through plants and seafood. Living in an area with low Se content in the soil exposes us to a high risk of Se-deficient blood.

Similarly, Ligowe et al. in 2019 established a geospatial linkage between the risk of Se deficiency and sub-Saharan food systems, mainly through limited access to animal sources. They reported that the Se level in individuals is typically dependent upon the region one lives in and the foods they consume. Dietary customs or habits may also influence Se intake. The population with limited accessibility to the best sources of Se (meat, fish, and eggs) may be at higher risk of Se deficiency [54].

Diets in Popokabaka are mainly based on cassava starch and leaves, which lack Se and selenoproteins (selenomethionine and selenocysteine). The consumption of such a staple food without significant animal food sources may limit the Se intake and its tissue stock. Se deficiency has been associated with some health outcomes. Ngo et al. [55] observed that Se deficiency was associated with iodine deficiency and cretinism in women with endemic myxedematous goiter in DRC (ancient Zaire), including Popokabaka. Although iodine disorder diseases (IDDs) have been almost eliminated through the successful iodine–salt fortification strategy, the presence of a possible iodine deficiency should be considered in our study area. Popokabaka is an ancient endemic goiter area with unknown Se intervention. Despite IDDs, Bumoko et al. [23] found a lower serum Se level in children with Konzo disease and a significant association between Se deficiency and cognitive performance parameters. Konzo is also prevalent in the Popokabaka region [56,57,58,59] and has, like IDDs, been associated with cyanate precursors that remain highly concentrated in inappropriately processed cassava plants. Food-based strategies, which are most commonly recommended in the case of population Se deficiency, should consider the cyanate metabolic patterns that are implicated these health outcomes. Mass Se supplementation, observed as a marginal strategy because of its overdose consequences, should not be recommended in this case.

### 4.4. Limitations and Strengths

Our cross-sectional study identified some factors as possible determinants of serum Zn, Cu, and Se levels among children in Popokabaka. However, temporal relationships between these factors (exposures) and outcome variables of interest cannot be ensured; thus, causality cannot be confirmed. 

In addition to the representativeness of the population of Popokabaka children, our study has the strength of providing community-based information on the serum level of multiple essential TEs from this rural hard-to-reach area for the first time. Our results are generalizable to the larger child population of Popokabaka, and deficiencies we have reported might be expected for similar rural regions in DRC. Furthermore, repeatable handling procedures were tested to manage contamination risk and cold chain challenges in such complex research conditions.

## 5. Conclusions

Mineral levels in the body depend on the population’s characteristics and its diet, geographic area, and soil composition. We report that the serum Se and Zn concentration in children under five years old, residing in Popokabaka, is significantly below standard cut-off values, thus indicating a severe dietary deficiency of these TEs. A cost-effective and sustainable intervention to address the Se and Zn status in Popokabaka now emerges as a research priority.

## Figures and Tables

**Table 1:** General characteristics of the population in a cross-sectional study on the micronutrient status in children from Popokabaka.

Characteristics	N (412)	%
Age [median (P25–P75)]	32 (22–43)	
Sex		
Boy	212	51.5
Girl	200	48.5
Stunting	228	55.3
Wasting	44	10.7
Underweight	140	34.0
Fever in the two preceding weeks	239	58.0
Diarrhea in the two preceding weeks	71	17.2
Cough in the two preceding weeks	133	32.3
Anemia	280	68.0
Iron deficiency	53	12.9
Iron-deficiency anemia	31	7.5
Inflammation state (elevated CRP)	202	49.0
Malaria by rapid test	358	86.9
Zinc supplementation in the two preceding weeks	21	5.1
Iron supplementation in the two preceding weeks	147	35.7
Micronutrient powders in the two preceding weeks	77	18.7

**Table 2:** Distribution of serum Zn, Cu, and Se as median (P25–P75) over different groups of children.

		*n*	Zn µg/dL	Cu µg/dL	Se µg/dL
**Total**		412	61.9(52.8–70.2)	145.5 (120.0–167.0)	5.3 (4.3–6.3)
**Child sex**					
	Male	212	61.8 (52.3–70.5)	150.0 (125.0–173.0)	5.2(4.1–6.3)
	Female	200	62.4 (52.9–70.1)	142.0 (116.0–162.5)	5.4 (4.4–6.3)
	*p*-value		0.816	0.093	0.403
**Age group**					
	12–23	117	61.7 (53.3–71.6)	146.0 (124.0–170.0)	5.4 (4.5–6.3)
	24–35	115	61.8 (52.8–69.0)	143.0 (113.0–166.0)	5.2 (4.1–5.9)
	36–47	110	63.3 (53.2–70.8)	151.5 (131.0–169.0)	5.3 (4.8–6.4)
	48–59	70	60.6 (52.0–71.0)	139.0 (110.0–163.0)	5.3 (3.7–6.2)
	*p*-value		0.826	0.099	0.442
**Anemia**					
	No	132	63.4 (54.2–73.0)	147.0 (118.5–164.5)	5.5 (4.7–6.4)
	Yes	280	60.9 (52.1–68.8)	145.0 (120.5–170.5)	5.2 (4.1–6.1)
	*p*-value		0.220	0.240	0.033
**Iron deficiency**					
	No	359	62.0 (53.7–70.7)	144.0 (117.0–166.0)	5.3 (4.3–6.3)
	Yes	53	60.1 (49.5–69.8)	150.0 (131.0–173.0)	5.5 (4.1–6.4)
	*p*-value		0.043	0.019	0.918
**Stunting**					
	No	184	62.5 (53.0–72.9)	146.0 (120.0–173.0)	5.4 (4.5–6.6)
	Yes	228	61.5 (52.7–68.4)	144.0 (119.0–164.0)	5.2 (3.9–6.0)
	*p*-value		0.293	0.456	0.004
**Inflammation state**					
	No	210	63.3 (56.7–71.9)	135.0 (111.0–155.0)	5.6 (4.8–6.7)
	Yes	202	59.5 (50.2–67.7)	155.5 (136.0 – 181.0)	5.0 (3.8–5.8)
	*p*-value		<0.001	<0.001	<0.001
**Malaria**					
	No	54	64.0 (55.1–75.5)	152.0 (133.0–173.0)	5.4 (4.6–5.9)
	Yes	358	61.4 (52.6–68.9)	145.0 (119.0–166.0)	5.3 (4.3–6.4)
	*p*-value		0.140	0.533	0.415
**Kwango river side**					
	East	343	60.2 (52.2–68.2)	146.0 (120.0–168.0)	5.2 (4.1–5.0)
	West	69	67.3 (61.9–74.8)	145.0 (119.0–163.0)	6.2 (5.2–7.0)
	*p*-value		<0.001	0.976	<0.001

**Table 3:** Prevalence of Zn, Cu, and Se deficiencies among 1–5-year-olds in Popokabaka.

	Unadjusted Prevalence % (95 CI)	CRP-Adjusted Prevalence % (95 CI)
**Zn deficiency**	64.6 (59.8–69.0)	64.6 (59.8–69.0)
**Cu deficiency**	1.5 (0.6–3.2)	1.5 (0.6–3.2)
**Se deficiency**	86.9 (83.3–89.8)	84.1 (81.4–87.0)

**Table 4:** Spearman correlation coefficient between continuous variables.

	Zinc	Copper	Selenium
Copper	0.23 ***	1.000	0.35 ***
Selenium	0.35 ***	0.07	1.000
Height-for-Age Z Score	0.07	0.05	0.15 **
Weight-for-Age Z Score	−0.05	−0.03	0.00
Household food-insecurity access score	−0.14 **	−0.09	0.05
Hemoglobin	0.14 **	−0.06	0.17 **
Transferrin saturation	−0.00	−0.12 **	−0.06
C-reactive Protein	−0.24 ***	0.39 ***	−0.33 ***

** *p*-value < 0.01; *** *p*-value < 0.001.

**Table 5:** Multiple linear regression analysis and β coefficients of determinants of serum zinc, copper, and selenium levels.

	Zn-Model				Cu-Model				Se-Model			
	Crude Estimates		Adjusted Estimates		Crude Estimates		Adjusted Estimates		Crude Estimates		Adjusted Estimates	
	β	95% CI	β	95% CI	Β	95% CI	β	95% CI	Β	95% CI	β	95% CI
**Sex girl**	1.55	[−2.07; 5.17]	-	-	−10.02 ^a^	[−17.47; −2.57]	−8.32 ^b^	[−15.37; −1.26]	0.08	[−0.22; 0.37]	-	-
**Age**	0.06	[−0.08; 0.21]	-	-	−0.32 ^a^	[−0.62; −0.03]	−0.26	[−0.54; 0.02]	0.00	[−0.01; 0.01]	-	-
**Diarrhea**	−4.50 ^a^	[−9.27; 0.28]	−4.36	[−9.11; 0.40]	−4.57	[−14.50; 5.36]	-	-	−0.19	[−0.58; 0.20]	-	-
**Zinc supplementation**	2.56	[−5.67; 10.78]	-	-	8.62	[−8.42; 25.68]	-	-	0.38	[−0.30; 1.04]	-	-
**Fever**	0.52	[−3.14; 4.19]	-	-	5.99 ^a^	[−1.60; 13.57]	1.93	[−5.32; 9.18]	−0.14	[−0.44; 0.16]	-	-
**Cough**	0.06	[−3.81; 3.93]	-	-	3.38	[−4.64; 11.40]	-	-	−0.26 ^a^	[−0.57; 0.06]	−0.21	[−0.50; 0.09]
**Iron supplementation**	−0.68	[−4.45; 3.10]	-	-	9.89 ^a^	[2.12; 17.67]	8.25 ^b^	[0.86; 15.63]	−0.06	[−0.37; 0.25]	-	-
**Micronutrient powder**	−1.45	[−6.09; 3.19]	-	-	−0.80	[−10.43; 8.82]	-	-	−0.08	[−0.46; 0.30]	-	-
**Elevated CRP**	−2.29	[−5.90; 1.32]	-	-	24.5 ^a^	[17.4; 31.64]	23.4 ^b^	[16.30; 30.45]	−0.76 ^a^	[−1.05; −0.47]	−0.68 ^b^	[−0.95; −0.40]
**Height-for-Age Z Score**	−0.27	[−0.82; 1.35]	-	-	0.37	[−1.87; 2.62]	-	-	0.13 ^a^	[0.04; 0.22]	0.12 ^b^	[0.04; 0.20]
**Weight-for-Age Z Score**	−1.02 ^a^	[−2.45; 0.42]	−0.94	[−2.37; 0.48]	−0.56	[−3.54; 2.42]	-	-	−0.04	[−0.15; 0.08]	-	-
**HFIAS**	−0.27 ^a^	[−0.55; 0.02]	−0.25	[−0.53; 0.04]	−0.38	[−0.97; 0.22]	-	-	0.00	[−0.02; 0.03]	-	-
**Malaria**	−1.89	[−7.25; 3.47]	-	-	−5.57	[−16.68; 5.54]	-	-	0.15	[−0.29; 0.59]	-	-
**Hgb**	−0.04	[−0.46; 0.38]	-	-	−0.34	[−1.21; 0.54]	-	-	0.00	[−0.03; 0.04]	-	-
**Transferrin saturation**	0.02	[−0.02; 0.05]	-	-	−0.08 ^a^	[−0.15; −0.02]	−0.08 ^b^	[−0.14; −0.02]	0.00	[−0.00; 0.00]	-	-
**Western side river**	6.20 ^a^	[1.39; 11.0]	5.93 ^b^	[1.13; 10.74]	−2.95	[−13.00; 7.10]	-	-	1.13 ^a^	[0.76; 1.52]	1.04 ^b^	[0.67; 1.41]

^a^*p*-value < 0.20 for inclusion in the model; ^b^ *p*-value < 0.05 for determinant significance.

## Data Availability

The dataset of this study can be made available on reasonable request to B.K.M.
